# Women’s reproductive health decision-making: A multi-country analysis of demographic and health surveys in sub-Saharan Africa

**DOI:** 10.1371/journal.pone.0209985

**Published:** 2019-01-09

**Authors:** Eugene Kofuor Maafo Darteh, Kwamena Sekyi Dickson, David Teye Doku

**Affiliations:** 1 Department of Population and Health, University of Cape Coast, Cape Ghost, Ghana; 2 University of Tampere, Faculty of Social Sciences, Health Sciences and PERLA (Tampere Centre for Childhood, Youth and Family Research), University of Tampere, Tampere, Finland; USC Keck School of Medicine, Institute for Global Health, UNITED STATES

## Abstract

**Introduction:**

Women’s ability to make decisions regarding their reproductive health has important implications for their health and well-being. We studied the socio-demographic factors affecting reproductive health decision-making among women in 27 sub-Sahara African countries.

**Materials and methods:**

The study made use of pooled data from current Demographic and Health Survey (DHS) conducted from January 1, 2010 and December 31, 2016 in 27 countries in sub-Sahara African. Binary and multivariate logistic regression models were used to investigate the associations of women’s socio-demographic factors with decision-making regarding sexual reproductive health.

**Results:**

The proportion of women who can ask their partners to use a condom during sexual intercourse ranged from lowest in Mali (16.6%) to highest in Namibia (93.4%). Furthermore, the proportion of women who can refuse sex ranged from 18.3% in Mali to 92.4% in Namibia. Overall, approximately every five out of ten women can ask their partners to use a condom, six out ten women could refuse their partners sex and seven out of ten women could make at least 1 decision. Women from rural areas (OR = 0.51, CI = 0.48–0.54), those with no education (OR = 0.11, CI = 0.10–0.12), Muslim women (OR = 0.29, CI = 0.27–0.31), women not working (OR = 0.53, CI = 0.51–0.56) and women whose partners had no education (OR = 0.17, CI = 0.16–0.19) were less likely to make a decision on their reproductive health.

**Conclusion:**

Residence, age, level of education, religion, occupation and partner’s education were found to be associated with women’s decision-making about sexual intercourse, condom use and reproductive health decision-making index. This study contributes to the discourse on reproductive health decision-making in Africa. Policies and intervention targeted at improving women’s autonomy and empowering them to take charge of their sexual and reproductive health issues should be focused on these factors.

## Introduction

Sexual and reproductive health may refer to individuals being able to freely make decisions about their sexual activity, that is, choose when, where, and with whom to have sexual intercourse [1]. Reproductive health and rights of women became of global interest after the International Conference on Population Development (ICPD) Program of Action in Cairo in 1994. A total of 179 countries at the ICPD in Cairo advocated that women should have the rights to freely decide on their reproductive health devoid of any discrimination [1]. This was further affirmed by the fourth women conference in Beijing in 1995 [2].

Gender inequalities have effects on reproductive health decision-making and it is a principal component of the social context of reproductive health [3, 4]. Women’s ability to make decision regarding their reproductive health had been curbed due to their lack of autonomy and power relations with men being more influential in decision-making [4]. Women’s autonomy is critical for the social, economic and sustainable development of any country. In view of this, the sustainable development goal (SDG) emphasizes gender equality. It increases their reproductive control, attitudes and ability to negotiate for safer sex [5,6].

In the global combat against HIV and AIDS, studies had highlighted the essence of communication between sexual partners concerning safer sex [7,8]. Negotiation between sexual partners about condom use and safe sex is associated with increased use of condoms [9]. Studies have shown that women’s autonomy is repeatedly constrained by educational, economic, cultural and social conditions [10,11,12,13].

In low- and middle-income countries, particularly sub-Saharan Africa and Southern Asia, there are several socio-economic and cultural factors which affect women’s ability to make decision regarding their own health and those of their dependents, especially children [14,15]. Earlier studies have focused on some aspects of reproductive health, women autonomy and decision-making, using sub-populations within some countries in sub-Saharan Africa [5,10,16,17,18,19,20]. As far as we know, no study has investigated the subject in multiple countries in the region. We aim to provide national and regional estimates of women’s autonomy in reproductive health decision-making in 27 sub-Saharan African countries and we further examine the factors associated with reproductive health decision-making in these countries.

The concept of decision-making can be categorized in three main ideas, thus, decision making as a right [21], as a choice [22] and as a process [23]. Decision making is a process that starts with problem identification, data collection and gathering, analysis of data, findings, selecting an appropriate and most suitable solution from the alternative solutions and finally evaluation of the process [23]. Socio-demographic characteristics such as level of education [5], partner educational level [5, 10] and wealth status [10, 16] have the potential of influencing an individual in making informed decision. Exposure to information and knowledge and the ability to provide for one’s needs is a positive influence on decision-making. By extension, a woman’s decision making about her sexual and reproductive health will be effectively accomplished based on the ability to provide for her needs and her exposure to knowledge. Nonetheless, there are possibilities of other background characteristics such as religious affiliation [10,16], place of residence [23] and cultural leanings [18, 19, 20] that can have impact on the decision-making process [15]. In sub-Saharan Africa in particular, religious affiliation, cultural and social and place of residence settings also affect women’s reproductive health decision-making [10]. This argument is consistent with the Pred’s Behavioural Matrix [23].

## Materials and methods

The study made use of pooled data from current Demographic and Health Survey (DHS) conducted from January 1, 2010 and December 31, 2016 in 27 countries in sub- Saharan Africa. DHS is a nationwide survey collected every five-year period across low and middle-income countries. DHS focuses on maternal and child health by interviewing women of reproductive age (15–49 years). DHS surveys follow the same standard procedures—sampling, questionnaires, data collection, cleaning, coding and analysis—which allows for cross–country comparison. The survey employs a stratified two-stage sampling technique. The first stage involved the selecting of points or clusters (enumeration areas [EAs]). The second stage is the systematic sampling of households listed in each cluster or EA. All women in their reproductive age (15–49) who were usual of selected households or visitors who slept in the households on the night before the survey were interviewed. The response rate varied from 86.2% to 100.0%. For the purpose of this, only women who had information on reproduction health decision-making were used (N = 210,536), thus, women who were either married or living with a partner. Women gave oral and written consent. Ethical approval for DHS is usually obtained from the ethics regulatory boards of the countries for which the studies are conducted and by ICF International’s institutional review board. Permission to use the data set was sort from MEASURE DHS. Data set is available to the public at https://dhsprogram.com/data/available-datasets.cfm (data was not collected or owned by the authors; potential users would be given access once a concept note is sent to MEASURE DHS)

### Definition of variables

#### Outcome variables

The three main outcome variables used were: (1) decision-making on sexual intercourse, (2) decision-making on condom use, and (3) reproductive health decision-making index. For the first variable, women were asked if they can refuse their partner sex. For the second variable (i.e. decision-making on condom use), women were asked if the can ask their partners to use condoms. The response category of these variables were: 1 = “yes”, 2 = “no” and 3 = “don’t know/ not sure”. This response was categorized as 0 = “no and don’t know” and 1 = “yes” (see Darteh et al. [10]). The third outcome variable, reproductive health decision-making index, is generated from the combination of the decision-making on sexual intercourse and the decision-making on condom use variables. This was categorized as 0 = no decision and 1 = at least 1 decision.

#### Explanatory variables

The explanatory variables consist of: residence, age, wealth status, education, religion, occupation and partner’s education. Residence was categorized as urban and rural. Age was grouped in 5 –year interval: 15–19, 20–24, 25–29, 30–34, 35–39, 40–44, 45–49. Wealth status was derived from the ownership of a variety of household assets and categorized as poorest, poorer, middle, richer and richest. Level of education and partner’s education was captured as no education, primary, secondary and higher education. Religion was recorded as Christian, Muslims and Others. Religion was not available for Niger. Occupation was categorized as not working, working.

### Statistical analysis

All data sets from the 29 countries downloaded from MEASURE DHS were merged and appended as one data set before the analysis was done. Descriptive and inferential statistics were used. Descriptive figures are reported in percentages by countries. Binary and multivariate logistic regression models were used to investigate the relationship between the explanatory variables and the outcome variables. Two models were used to assess the predictors of women’s decision-making on sexual intercourse, decision-making on condom use, and reproductive health decision-making index. Model I looked at a bivariate analysis between each of the predictor variables and the outcome variable. Model II was fitted to investigate the association between the independent variables and the outcome variables (decision-making on sexual intercourse, decision making on condom use, and reproductive health decision-making index). All frequency distributions were weighted whilst the survey command in Stata was used to adjust for the complex sampling structure of the data in the regression analyses. All results of the logistic analyses were presented as odds ratios (ORs) with 95% confidence intervals (CIs).

## Results

Table 1 shows selected information from women from 27 countries in sub-Saharan Africa. The proportion of women who could ask their partner to use a condom during sexual intercourse ranged from 16.6% in Mali to 93.4% in Namibia. Proportion of women who could refuse sex ranged from 18.3% in Mali to 92.4% in Namibia. The proportion of women who could make at least 1 of the reproductive health decisions ranged from 25.5% in Mali to 97.2% in Namibia. Overall, approximately every five out of ten women could ask a partner to use a condom, six out ten women could refuse their partners sex and seven out of women could make at least 1 decision.

**Table 1:** Background characteristics of sampled women in 27 sub–Saharan African countries.

Country	Weighted N*	% can ask partner to use a condom		% can refuse sex		Reproductive health decision index	
		No	Yes	No	Yes	No decision	At least 1
Benin, 2011–2012	11,045	61.6	38.4	47.2	52.8	41.9	58.1
Burundi, 2011	5,424	33.7	66.3	39.4	60.6	19.6	80.4
Cameroon, 2011	4,682	42.7	57.3	25.4	74.6	19.9	80.1
Chad, 2014–2015	4,553	82.6	17.4	55.6	44.4	51.8	48.2
Comoros, 2012	3,087	54.4	45.6	55.7	44.3	43.5	56.5
Congo, 2011–2012	6,259	34.4	65.6	29.4	70.6	14.9	85.1
Congo DR, 2013–2014	12,119	60.1	39.9	33.8	66.2	25.8	74.2
Cote d’voire, 2011–2014	6,290	58.0	42.0	42.9	57.1	35.3	64.7
Ethiopia, 2011	10,225	63.2	36.8	47.0	53.0	38.0	62.0
Gabon, 2012	4,383	18.2	81.8	18.4	81.6	8.3	91.7
Gambia, 2013	6,763	55.7	44.3	47.7	52.3	39.6	60.4
Ghana, 2014	5,382	33.3	66.7	26.1	73.9	17.8	82.3
Guinea, 2012	6,727	75.8	24.2	59.9	40.1	39.6	60.4
Kenya, 2014	8,679	25.6	74.4	25.7	74.3	14.3	85.7
Lesotho, 2014–2015	1,662	7.9	92.1	24.6	75.4	3.2	96.8
Liberia, 2013	5,382	43.7	56.3	15.4	84.6	12.7	87.3
Malawi, 2010	15,577	22.4	77.6	25.1	74.9	12.3	87.7
Mali, 2012–2013	7,509	83.4	16.6	81.7	18.3	74.5	25.5
Mozambique, 2011	9,337	61.1	38.9	51.2	48.8	44.9	55.1
Namibia, 2015	3,090	6.6	93.4	7.7	92.3	2.8	97.2
Nigeria, 2013	27,749	61.9	38.1	38.7	61.3	33.7	66.3
Rwanda, 2014–2015	6,962	16.7	83.3	17.3	82.7	6.9	93.1
Sierra Leone, 2013	10,800	67.4	32.6	31.4	68.6	28.8	71.2
Senegal, 2010–2011	10,390	69.9	30.1	71.0	29.0	58.2	41.8
Togo, 2013–2014	6,298	39.8	60.2	28.1	71.9	21.7	78.3
Zambia, 2013–2014	9,837	24.2	75.8	30.4	69.6	15.4	84.6
Zimbabwe, 2015	6,155	29.2	70.8	28.2	71.8	14.1	85.9
All Countries (total)	210,536	50.2	49.8	39.3	60.7	30.7	69.3

*Number of women with data on reproductive health decision

Table 2 presents the odds of making a decision on sexual intercourse, condom use and reproductive health among women in 29 sub–Saharan African countries. Residence, age, wealth status, education, religion, occupation and partner’s education strongly predicted decision-making on sexual intercourse and condom use among women in the 29 sub–Saharan African countries. Women from rural areas were less likely to decide on sexual intercourse (OR = 0.58, CI = 0.55–0.61), condom use (OR = 0.54, CI = 0.51–0.57) and overall decisions on reproductive health (OR = 0.51, CI = 0.48–0.54), compared to those from urban areas. The effects of women’s age on their ability to take decision varied and were mainly positive. For instance, women aged 20–24 years were 1.07 times more likely to decide to have sex or not (CI = 1.02–1.12), 1.54 times more likely to decide to use condom or not (CI = 1.47–1.62) and 1.20 times more likely to decide on their reproductive health (CI = 1.14–1.26), compared to women aged 15–19 years. The relationship between wealth status and women’s ability to make decision was mainly negative. Women with poorest wealth status were 0.44 times less likely to decide to have sex or not, 0.32 times less likely to decide to use condom or not and 0.33 times less likely to decide on their reproductive health, compared to women with richest wealth status.

**Table 2:** Logistic regression model showing the relationship (Odds ratio) between background characteristics and women’s decision-making on sexual intercourse, condom use and reproductive health decision-making index in 27 sub-Saharan African countries, 2010–2016.

Explanatory factors	Proportions N = 210,536	Decision-making on sexual intercourse	Decision-making on condom use	Reproduction health decision-making index
		Model I Odds Ratio (OR) 95% CI	Model I Odds Ratio (OR) 95% CI	Model Odds Ratio (OR) 95% CI
***Residence***				
Urban	73,097	Ref	Ref	Ref
Rural	137,439	0.58***(0.55–0.61)	0.54***(0.51–0.57)	0.51***(0.48–0.54)
***Age***				
15–19	16,259	0.75***(0.70–0.79)	1.05***(0.98–1.11)	0.79***(0.74–0.84)
20–24	39,968	1.07**(1.02–1.12)	1.54***(1.47–1.62)	1.20***(1.14–1.26)
25–29	51,524	1.11***(1.06–1.16)	1.54***(1.47–1.62)	1.23***(1.17–1.29)
30–34	43,210	1.14***(1.09–1.19)	1.49***(1.43–1.56)	1.25***(1.19–1.31)
35–39	36,717	1.10***(1.05–1.16)	1.39***(1.33–1.46)	1.19***(1.32–1.25)
40–44	25,567	1.04(0.99–1.09)	1.21***(1.15–1.28)	1.08**(1.03–1.14)
45–49	20,075	Ref	Ref	Ref
***Wealth status***				
Poorest	42,002	0.44***(0.42–0.47)	0.32***(0.30–0.34)	0.33***(0.31–0.35)
Poorer	42,981	0.53***(0.50–0.56)	0.41***(0.39–0.44)	0.42***(0.39–0.45)
Middle	41,850	0.58***(0.55–0.62)	0.49***(0.46–0.51)	0.47***(0.45–0.51)
Richer	42,003	0.72***(0.68–0.75)	0.64***(0.60–0.67)	0.63***(0.59–0.67)
Richest	41,680	Ref	Ref	Ref
***Educatio****n*				
No education	88,230	0.19***(0.17–0.21)	0.12***(0.11–0.13)	0.11***(0.10–0.12)
Primary	66,581	0.45***(0.41–0.49)	0.40***(0.36–0.43)	0.34***(0.30–0.38)
Secondary	47,764	0.65***(0.60–0.72)	0.56***(0.52–0.61)	0.56***(0.50–0.63)
Higher	7,961	Ref	Ref	Ref
***Religion***				
Muslims	76,621	0.39***(0.36–0.40)	0.30***(0.29–0.32)	0.29***(0.27–0.31)
Christians	121,473	Ref	Ref	Ref
Others	12,442	0.54***(0.50–0.59)	0.41***(0.38–0.45)	0.40***(0.37–0.44)
***Occupation***				
Not working	64,520	0.58***(0.56–0.60)	0.67***(0.65–0.70)	0.53***(0.51–0.56)
Working	146,016	Ref	Ref	Ref
***Partner’s education***				
No education	72,118	0.25***(0.23–0.27)	0.18***(0.17–0.19)	0.17***(0.16–0.19)
Primary	59,380	0.56***(0.53–0.61)	0.58***(0.55–0.62)	0.51***(0.47–0.55)
Secondary	62,269	0.80***(0.75–0.86)	0.73***(0.69–0.78)	0.75***(0.70–0.81)
Higher	16,769	Ref	Ref	Ref
Country				
Benin	11,045	Ref	Ref	Ref
Burundi	5,424	1.55***(1.44–1.65)	3.40***(3.18–3.65)	3.24***(3.00–3.50)
Cameroon	4,682	2.73***(2.53–9.94)	2.40**(2.24–2.57)	3.08***(2.84–3.34)
Chad	4,553	0.65***(0.61–0.70)	0.29***(0.27–0.32)	0.62***(0.57–0.66)
Comoros	3,087	0.76***(0.70–0.82)	1.56***(1.44–1.69)	1.05***(0.97–1.14)
Congo	6,259	1.80***(1.70–1.89)	1.00(0.94–1.05)	2.05***(1.95–2.17)
Congo DR	12,119	2.47***(2.31–2.63)	2.83***(2.66–3.02)	4.52***(4.18–4.89)
Cote d’voire	6,290	1.09**(1.03–1.16)	1.10**(1.04–1.18)	1.24***(1.16–1.32)
Ethiopia	10,225	1.06*(1.00–1.11)	0.98(0.92–1.03)	1.18***(1.11–1.24)
Gabon	4,383	4.55***(4.18–4.94)	6.55***(6.04–7.10)	7.74***(6.96–8.61)
Gambia	6,763	0.86***(0.81–0.91)	1.19***(1.12–1.26)	1.00(0.94–1.07)
Ghana	5,382	2.64***(2.46–2.84)	3.29***(3.08–3.53)	3.35***(3.10–3.62)
Guinea	6,727	0.66***(0.62–0.70)	0.58***(0.54–0.62)	0.69***(0.65–0.74)
Kenya	8,679	2.31***(2.18–2.45)	3.61***(3.40–3.83)	3.38***(3.17–3.61)
Lesotho	1,662	2.69***(2.38–3.04)	26.14***(21.07–32.43)	20.06***(15.29–26.33)
Liberia	5,382	4.79***(4.42–5.18)	2.12***(1.99–2.26)	4.90***(4.61–5.33)
Malawi	15,577	2.92***(2.78–3.08)	6.45***(6.11–6.81)	5.96***(5.60–6.33)
Mali	7,509	0.22***(0.21–0.23)	0.37***(0.34–0.39)	0.29***(0.27–0.31)
Mozambique	9,337	1.13***(1.06–1.19)	1.41***(1.33–1.49)	1.58***(1.51–1.65)
Namibia	3,090	9.99***(8.80–11.34)	21.60***(18.84–24.75)	22.30***(18.39–27.04)
Nigeria	27,749	1.55***(1.48–1.62)	1.06*(1.01–1.11)	1.58***(1.51–1.65)
Niger	9,866	0.44***(1.48–1.62)	0.55***(0.52–0.58)	0.54***(0.52–0.57)
Rwanda	6,962	4.46***(4.15–4.79)	8.45***(7.85–9.10)	10.36***(9.27–11.46)
Sierra Leone	10,800	2.11***(1.99–2.23)	0.93*(0.88–0.98)	1.94***(1.83–2.05)
Senegal	10,390	0.34***(0.32–0.37)	0.64***(0.60–0.67)	0.49***(0.46–0.51)
Togo	6,298	2,28***(2.01–2.60)	2.10***(1.97–2.23)	2.28***(2.13–2.44)
Uganda	7,125	3.31***(3.10–3.54)	2.92***(2.74–3.10)	4.20***(3.90–4.52)
Zambia	9,837	2.28***(2.16–2.42)	5.72***(5.38–6.08)	4.58**(4.28–4.90)
Zimbabwe	6,155	2.50***(2.34–2.67)	4.37***(4.09–4.68)	4.78***(4.40–5.19)

*p < 0.05

** p < 0.01

***p < 0.001

The relationship between religion and women’s ability to make decision was mainly negative. Muslim women were 0.39 times less likely to decide to have sex or not, 0.30 times less likely to decide to use condom or not and 0.29 times less likely to decide on on their reproductive health, compared to Christian women. Women who were not working were 0.58 times less likely to decide to have sex or not, 0.69 times less likely to decide to use condom or not and 0.54 times less likely to decide on their reproductive health, compared to women working outside their home. On the other hand, women who work at home were 1.33 times more like to decide to have sex or not, 2.56 times more likely to decide to use condom or not and 1.89 times more likely to decide on their reproductive health, compared to women working outside their home. Women with no education were 0.19 times less likely to decide to have sex or not, 0.12 times less likely to decide to use condom or not and 0.11 times less likely to decide on their reproductive health, compared to women with higher education.

In the final multivariate model, the statistical associations between all the exposures and the three outcomes were retained, although most of the odds were attenuated (Table 3). The only exception is that the associations of decision-making on sexual intercourse with age and wealth status lost their statistical significance.

**Table 3:** Multivariate logistic regression model showing the relationship (Adjusted odds ratio) between background characteristics and women’s decision-making on sexual intercourse, condom use and reproductive health decision-making index in 27 sub–Saharan African countries, 2010–2016.

Explanatory factors	Proportions N = 210,536	Decision-making on sexual intercourse	Decision-making on condom use	Reproduction health decision-making index
		Model II Adjusted Odds Ratio (AOR) 95% CI	Model II Adjusted Odds Ratio (AOR) 95% CI	Model II Adjusted Odds Ratio (AOR) 95% CI
***Residence***				
Urban	73,097	Ref	Ref	Ref
Rural	137,439	0.89***(0.86–0.91)	0.83***(0.81–0.85)	0.81***(0.81–0.86)
*Age*				
15–19	16,259	0.93***(0.88–0.97)	1.35***(1.28–1.41)	0.99(0.94–1.05)
20–24	39,968	1.04*(0.99–1.07)	1.53***(1.46–1.59)	1.14***(1.10–1.20)
25–29	51,524	1.04**(1.00–1.08)	1.49***(1.44–1.66)	1.16***(1.11–1.20)
30–34	43,210	1.06**(1.02–1.10)	1.41***(1.36–1.47)	1.15***(1.03–1.20)
35–39	36,717	1.02(0.98–1.06)	1.32***(1.27–1.38)	1.10***(1.05–1.14)
40–44	25,567	0.99(0.95–1.03)	1.16***(1.11–1.21)	1.02(0.97–1.07)
45–49	20,075	Ref	Ref	Ref
***Wealth status***				
Poorest	42,002	0.74***(0.71–0.77)	0.59***(0.57–0.62)	0.61***(0.58–0.64)
Poorer	42,981	0.80***(0.76–0.83)	0.69***(0.66–0.72)	0.69***(0.66–0.72)
Middle	41,850	0.82***(0.78–0.85)	0.74***(0.71–0.77)	0.72***(0.69–0.75)
Richer	42,003	0.88***(0.95–1.03)	0.81***(0.78–0.84)	0.81**(0.77–0.84)
Richest	41,680	Ref	Ref	Ref
***Educatio****n*				
No education	88,230	0.49***(0.45–0.53)	0.34***(0.32–0.37)	0.37***(0.34–0.41)
Primary	66,581	0.66***(0.61–0.71)	0.52***(0.48–0.55)	0.54***(0.50–0.51)
Secondary	47,764	0.83***(0.77–0.88)	0.72***(0.67–0.77)	0.78***(0.71–0.85)
Higher	7,961	Ref	Ref	Ref
***Religion***				
Muslims	76,621	0.60***(0.59–0.62)	0.76***(0.74–0.78)	0.60***(0.58–0.62)
Christians	121,473	Ref	Ref	Ref
Others	12,442	0.89***(0.85–0.93)	0.82***(079–0.85)	0.84***(0.81–0.88)
***Occupation***				
Not working	64,520	0.78***(0.76–0.80)	0.82***(0.80–0.83)	0.72***(0.70–0.74)
Working	146,016	Ref	Ref	Ref
***Partner’s education***				
No education	72,118	0.74***(0.70–0.78)	0.60***(0.57–0.63)	0.65***(0.61–0.68)
Primary	59,380	0.92**(0.88–0.97)	0.75***(0.71–0.79)	0.84***(0.79–0.89)
Secondary	62,269	0.99(0.95–1.04)	0.91***(0.87–0.95)	0.95(0.90–1.01)
Higher	16,769	Ref	Ref	Ref
Country				
Benin	11,045	Ref	Ref	Ref
Burundi	5,424	1.20***(1.12–1.30)	2.88***(2.68–3.10)	2.48***(2.28–2.69)
Cameroon	4,682	2.01***(1.85–2.18)	1.52***(1.40–1.64)	2.10***(1.92–2.29)
Chad	4,553	0.86***(0.80–0.93)	0.33***(0.30–0.35)	0.82***(0.76–0.88)
Comoros	3,087	0.86**(0.79–0.94)	1.26***(1.15–1.39)	1.06(0.97–1.16)
Congo	6,259	1.10**(1.04–1.16)	0.54***(0.51–0.57)	1.10**(1.04–1.17)
Congo DR	12,119	1.45***(1.35–1.56)	1.68***(1.56–1.80)	2.42***(2.22–2.64)
Cote d’voire	6,290	1.18***(1.11–1.26)	1.10**(1.03–1.18)	1.32***(1.23–1.41)
Ethiopia	10,225	1.15***(1.09–1.22)	0.96(0.90–1.02)	1.30***(1.23–1.38)
Gabon	4,383	2.79***(2.54–3.06)	3.64***(3.33–3.98)	4.12***(3.67–4.63)
Gambia	6,763	1.19***(1.11–1.27)	1.30***(1.21–1.39)	1.35***(1.26–1.45)
Ghana	5,382	1.94***(1.80–2.09)	2.25***(2.09–2.42)	2.29***(2.11–2.49)
Guinea	6,727	0.92*(0.86–0.99)	0.68***(0.63–0.73)	0.95(0.87–1.01)
Kenya	8,679	1.60***(1.50–1.71)	2.34***(2.20–2.50)	2.22***(2.06–2.49)
Lesotho	1,662	1.29***(1.14–1.47)	12.28***(9.83–15.32)	8.18***(6.19–10.81)
Liberia	5,382	4.06***(3.74–4.41)	1.73***(1.61–1.85)	4.05***(3.71–4.42)
Malawi	15,577	2.09***(2.65–3.09)	4.58***(4.31–4.87)	4.00***(3.74–4.28)
Mali	7,509	0.34***(0.32–0.37)	0.46***(0.43–0.50)	0.44***(0.41–0.47)
Mozambique	9,337	2.09***(1.97–2.21)	1.02(0.95–1.09)	0.89***(0.84–0.95)
Namibia	3,090	5.98***(5.23–6.83)	12.40***(9.83–15.32)	12.32***(10.05–15.10)
Nigeria	27,749	1.52***(1.45–1.60)	0.78***(0.74–0.82)	1.49***(1.41–1.57)
Rwanda	9,866	2.86***(2.65–3.09)	5.73***(5.29–6.20)	6.12***(5.51–6.80)
Sierra Leone	6,962	2.73***(2.557–2.90)	0.99(0.93–1.05)	2.45***(2.30–2.61)
Senegal	10,800	0.55***(0.52–0.59)	0.84***(0.79–0.89)	0.78***(0.73–0.83)
Togo	10,390	1.67***(1.56–1.79)	1.70***(1.59–1.82)	1.83***(1.70–1.96)
Zambia	6,298	1.38***(1.30–1.47)	3.33***(3.12–3.56)	2.49***(2.31–2.68)
Zimbabwe	7,125	1.31***(1.22–1.41)	1.94***(1.80–2.09)	2.08***(1.90–2.27)

*p< 0.05

** p < 0.01

***p < 0.001

NB: Reproductive health decision-making index is the combination of decision-making on sexual intercourse and the decision-making on condom use

## Discussion

This paper examined the prevalence of reproductive health decision-making and the factors that determine women’s decision-making on reproductive health issues in 27 sub-Saharan African countries. The study found out that there were wide variations in the proportion of women making decisions on sexual intercourse (18%-92%), condom use (16%-93%) and reproductive health decision-making index (25%-97%) across the region.

The study found out that about 1 in 2 women could not request their partners to use condom whilst two out of five could not refuse their partners sexual intercourse when they request. Three out of ten women could not make decision regarding their reproductive health. Similar results were seen by Darteh and collegues in 2014 [10] They reported that one-fifth of sampled Ghanaian women could not refuse their partners’ request for sexual intercourse whilst one out of four could not demand the use of condoms by their partners. Residence, women’s education, religion, occupation and partners’ education were independently associated with both decision-making on sexual intercourse, condom use as well as the reproductive health decision-making index while age and wealth were independently associated with decision-making regarding condom use and the reproductive health index. This is evident in earlier works of Exavery et al. [5]; Rahman et al. [13], Hammed et al.[24] and Sujath and Reddy[6].

Older women were seen to be more likely to make decision on sexual intercourse, condom use and reproductive health decision-making index, compared to the younger ones. This finding affirms the study by Hameed et al. [24] and Darteh et al. [10] who found that women with increased age tend to get higher decision-making power. In most African countries, younger women are not expected to argue with older persons and are required to respect their opinions at all time. There are also traces of intergenerational sexual relationships and age mixing between younger women and older men in a sexual relationship. There is also evidence of early marriage by younger women which is being used as a strategy for economic survival [25].

Women with poorest, poorer, middle and richer wealth status were less likely to make decision on sexual intercourse, condom use and reproductive health decision-making index, compared to women with richest wealth status similar to those found by [5,10,18,21]. Wealth may lead to autonomy and independence and is related to occupation and education. Wealth could also be associated with self-esteem and confidence and this may have effects on the ability to make decision regarding reproductive health issues.

Women with secondary and lesser education were less likely to make decision on sexual intercourse, condom use and reproductive health decision-making index, compared to women with higher education. Also, women whose partners had no education, primary and secondary education were less likely to make decision on sexual intercourse, condom use and reproductive health decision-making index, compared to those with higher education. This is consistent with previous studies [5,10,16,20]. Education empowers women to be independent and equip them with the essential information regarding sexual intercourse and condom use that may be important for making decisions regarding their reproductive health isssues. How well a person is informed affects decsion making at every stage [26].

Ealier studies had confirmed that one’s religious affiliation may have some effects on the decision-making with regards to reproductive health. The study also found that Muslim women and women from other religions were less likely to make decision on sexual intercourse, condom use and reproductive health decision-making index, compared to Christian women. This finding is consistent with Darteh et al. [10] but goes contrary to that of Exavery et al. [5] who found out that Muslims were more likely to make decision on condom use is Tanzania. The possible explanation may be because in Islam, women are expected by their traditions to respect men and not to challenge their authority. They are expected to be submissive under the control of male This may explain why Muslim women are not likely to make decision on their reproductive health decision-making.

With respect to occupation, women’s likelihood of making decision varied. Women who were not working were less likely to make decision on sexual intercourse, condom use and reproductive health decision-making index, compared to women working outside home. Similar to education, women who are working may have power and resources and consequently independent in making decisions since they may not depend on their spouses for every resource compared to those who are not working. Women working are most likely literates and thus informed about their reproductive rights.

This study relied on cross–sectional design and hence causal inferences cannot be made. The study relied on self-reported measures which could be affected by social and cultural biases and subject to given desirable answers. Regardless of the shortfalls, the study has persuasive strengths. The DHS surveys have standardized questionnaires, sampling procedures and methodology which make data comparable among countries. The surveys in all countries were nationally representative and the response rates were high.

## Conclusion

Residence, age, level of education, religion, occupation and partner’s education were found to be associated with women’s decision-making about sexual intercourse, condom use and reproductive health decision-making index. Findings from the study provide robust evidence on factors associated with the reproductive health decision and contribute to the discourse on reproductive health decision-making in sub-Saharan Africa. Policies and intervention targeted at improving women autonomy and empowering women to take charge of their sexual and reproductive health issues should be focused on younger women, those from rural areas, those with secondary or less education attainment, those from less wealth background, those who are unemployed, Muslims and those with other religious belief.
